# How to Make Local Anesthesia Less Painful

**DOI:** 10.4103/0974-2077.41161

**Published:** 2008-01

**Authors:** Sharad Mutalik

**Affiliations:** *Consultant Dermatologist, 562/5, Shivajinagar, Near Congress Bhavan, Pune, Maharashtra, India*

**Keywords:** Local anaesthesia, Pain, Cutaneous Surgery, Lignocaine

## Abstract

Most cutaneous surgeries are performed under local anaesthesia. It is important the process of administration of local anaesthetics is pain free. This article suggests simple tips to make local anaestesia less painful.

## INTRODUCTION

Dermatologists have always been at the forefront of office-based surgery. Office-based surgery cannot be accomplished without the help of local anaesthesia. Local anaesthetic administration with intradermal or subcutaneous lignocaine infiltration is associated with discomfort. The level of pain felt during surgery is of utmost importance and has a significant impact on the patient. It is gratifying to hear at the end of a surgery, “Did you start yet?”

Number of injections, size of the needle, amount and type of anaesthetic, level of anxiety of the patient are all important factors which contribute to pain during local anaesthesia. Besides these factors, individual variations in reactions to pain do exist.

We have adopted the following techniques to reduce pain during local anaesthesia:

1. Sodium bicarbonate was added to the local anaesthetic solution. This reduces the stinging sensation caused by acidic pH (adrenaline). The formulation used was as follows:
  2% Lignocaine, 19 ml
  Adrenaline 1: 1000, 0.1 ml
  Normal saline, 20 ml
  Sodium bicarbonate (8.3%), 4 ml
  Eighty percent patients reported much reduced pain as compared to experience on the control site.
2. Bupivacaine was used when prolonged local anaesthesia (LA) was desired. Bupivacaine has more prolonged action and hence repeated pricks are not necessary.
3. Other measures to reduce the needle prick pain, include, pre-cooling with ice packs, use of eutectic mixture of local anaesthetic (EMLA) cream, 4% xylocaine ointment, 10% xylocaine spray, ethyl chloride spray, Mucopain^®^ or xylocaine viscus for mucosa, use of small bore needle 27G, 30G.
4. *Anxiety alleviation*: Apprehensive patients were given premedication with anxiolytic like diazepam or alprazolam. For very anxious patients conscious sedation was induced using midazolam, pentazocine or propofol. Both the approaches made it easier to give needle pricks in majority of patients.
5. A useful modification is to give the first prick intradermally, at right angles to the surface and a small quantity infiltrated to raise a wheal. After waiting for 2 min subsequent prick were given through the anaesthetized area. Repetitive rapid rubbing and shaking of the skin proximal to the site of injection during lignocaine infiltration also reduced the level of pain and discomfort.

## DISCUSSION

Reasons for pain during administration of local anaesthesia include needle prick, acidic medium of the medication and improper technique. Addition of sodium bicarbonate reduced the stinging sensation related to the acidic nature of adrenaline containing LA. Similarly various topical applications prior to prick also helped patients to tolerate the prick pain better. Apprehension is always a big problem in any surgical procedure. Conscious sedation utilizes a combination of sedatives, analgesics and tranquilizers to induce a state of amnesia, anxiolysis and analgesia. This places the patient in a quiescent state so that LA and nerve blocks may be comfortably administered.

Reduction in the level of pain and discomfort by repetitive rapid pinching and shaking of the skin proximal to the site of injection during lignocaine infiltration works on the ‘gate control hypothesis’: the ascending transmission of pain by thin slow fibres could be modified or “*gated*” at the spinal level by afferent signals carried by thick fast fibres emanating from the same dermatome. When the low-threshold fibres are activated by pressure and/or vibration, this stimulus transmission *gates* or diminishes the pain stimulus, reducing the perception of pain.

Finally, it is important to make the patient comfortable during any office surgery. If the patient is uncomfortable, he/she is more likely to move during surgery, which can make the surgeon uncomfortable, leading to detrimental outcome.

